# Electrospun Poly(L-Lactide) Fiber with Ginsenoside Rg3 for Inhibiting Scar Hyperplasia of Skin

**DOI:** 10.1371/journal.pone.0068771

**Published:** 2013-07-18

**Authors:** Wenguo Cui, Liying Cheng, Changmin Hu, Haiyan Li, Yuguang Zhang, Jiang Chang

**Affiliations:** 1 School of Biomedical Engineering and Med-X Research Institute, Shanghai Jiao Tong University, Shanghai, The People’s Republic of China; 2 Department of Plastic and Reconstructive Surgery, Ninth People’s Hospital affiliated to Medical School of Shanghai Jiao Tong University, Shanghai, The People’s Republic of China; 3 State Key Laboratory of High Performance Ceramics and Superfine Microstructure, Shanghai Institute of Ceramics, Chinese Academy of Sciences, Shanghai, The People’s Republic of China; The Ohio State University, United States of America

## Abstract

Hypertrophic scarring (HS) has been considered as a great concern for patients and a challenging problem for clinicians as it can be cosmetically disfiguring and functionally debilitating. In this study, Ginsenoside Rg3/Poly(l-lactide) (G-Rg3/PLLA) electrospun fibrous scaffolds covering on the full-thickness skin excisions location was designed to suppress the hypertrophic scar formatio*n in vivo*. SEM and XRD results indicated that the crystal G-Rg3 carried in PLLA electrospun fibers was in amorphous state, which facilitates the solubility of G-Rg3 in the PLLA electrospun fibrous scaffolds, and solubility of G-Rg3 in PBS is increased from 3.2 µg/ml for pure G-Rg3 powders to 19.4 µg/ml for incorporated in PLLA-10% fibers. The released G-Rg3 content in the physiological medium could be further altered from 324 to 3445 µg in a 40-day release period by adjusting the G-Rg3 incorporation amount in PLLA electrospun fibers. *In vitro* results demonstrated that electrospun G-Rg3/PLLA fibrous scaffold could significantly inhibit fibroblast cell growth and proliferation. *In vivo* results confirmed that the G-Rg3/PLLA electrospun fibrous scaffold showed significant improvements in terms of dermis layer thickness, fibroblast proliferation, collagen fibers and microvessels, revealing that the incorporation of the G-Rg3 in the fibers prevented the HS formation. The above results demonstrate the potential use of G-Rg3/PLLA electrospun fibrous scaffolds to rapidly minimize fibroblast growth and restore the structural and functional properties of wounded skin for patients with deep trauma, severe burn injury, and surgical incision.

## Introduction

Hypertrophic scarring (HS) is a dermal fibroproliferative disorder that often occurs following deep trauma, severe burn injury, and surgical incision [1], and is a great concern for patients and a challenging problem for clinicians [2]. HS is normally characterized by proliferation of dermal tissue with excessive deposition of fibroblast-derived extracellular matrix proteins, especially collagen, by excessive neovascularization, persistent inflammation, and fibrosis [3]. Some studies have shown that HS growth can be inhibited by reducing the growth of scar fibroblast proliferation or inducing apoptosis of fibroblasts to reduce collagen synthesis and secretion [4], [5]. Therefore, selective inhibition of excessive proliferation of fibroblasts in early proliferative phase of HS is a promising new method for treating HS.

Ginsenoside Rg3 (G-Rg3), a saponin, extracted from Red Panax ginseng, is a very powerful angiogenic inhibitor [6], [7]. Some findings demonstrated that G-Rg3 reduced generation of new blood capillaries and excessive vascular formation by inhibiting vascular endothelial cell proliferation and vascular endothelial growth factor expression, which further inhibited HS formation [7]–[9]. In addition, G-Rg3 was also found to be able to induce apoptosis of human hypertrophic scar fibroblasts [10], [11]. Therefore, we speculate that G-Rg3 may be applied on the injury tissue for preventing or reducing HS formation.

However, it is very critical to find a proper drug delivery system for G-Rg3 due to its poor solubility and high crystallinity under physiological conditions. Some studies reported that low loading efficiency and burst release were observed in microcapsules, which indicates that it is challenging to regulate the release rate of G-Rg3 in order to maintain suitable drug concentration within a therapeutic window for enough exposure time [12]–[15]. The main formulation of G-Rg3 medications is a mixture suspension of G-Rg3 and physiological saline [16], [17]. However, i*n vivo* study showed that the absorptivity of G-Rg3 was very low and most of G-Rg3 was metabolized due to its low solubility and high aggregation when the G-Rg3 suspension was injected into body, which limited G-Rg3’s efficiency [18]. Other studies have been conducted to solve this problem using different drug delivery vehicles for G-Rg3, such as spray-drying particles and microcapsules [19]. However, these studies revealed that the encapsulation and utilization efficiencies of G-Rg3 were very low because of its agglomeration and poor solubility [20]. Thus, the preparation of an effective delivery system for G-Rg3 is very critical. This delivery system should be able to avoid G-Rg3’s agglomeration, increase its solubility in physiological environment and improve its absorption by host. Furthermore, we expect to achieve a delivery system which can avoid its burst release once the drug delivery vehicles are applied *in vivo*.

Fibrous pharmaceutical formulation is one of the local sustained drug release system, and electrospinning has been reported as a potential technique for fabricating polymeric micro/nano-fibrous pharmaceutical formulations [21]–[23]. Electrospun fibers, with the diameter between 50 nm and 50 µm, have shown many outstanding properties, such as large surface area, high length–diameter ratio, flexible surface functionalities, tunable surface morphologies and proper mechanical performance. Electrospun fibrous mats consisting of ultrafine fibers have been widely used as scaffolds for tissue engineering, substitutes for tissue repair, materials for wound dressing, and carriers for drug delivery [24]. Electrospun fibrous mats have many advantages for drug delivery. As the electrospinnig process is very mild, conventional drugs, proteins and even DNA can be uniformly entrapped into fibers without denaturation and inactivation of drugs [25], [26]. In addition, drug release behavior can be controlled by adjusting fibrous morphology, structure and composition. Furthermore, electrospun fibrous scaffolds can be cut into any shape, which is very convenient for clinical applications [27].

**Figure 1:**
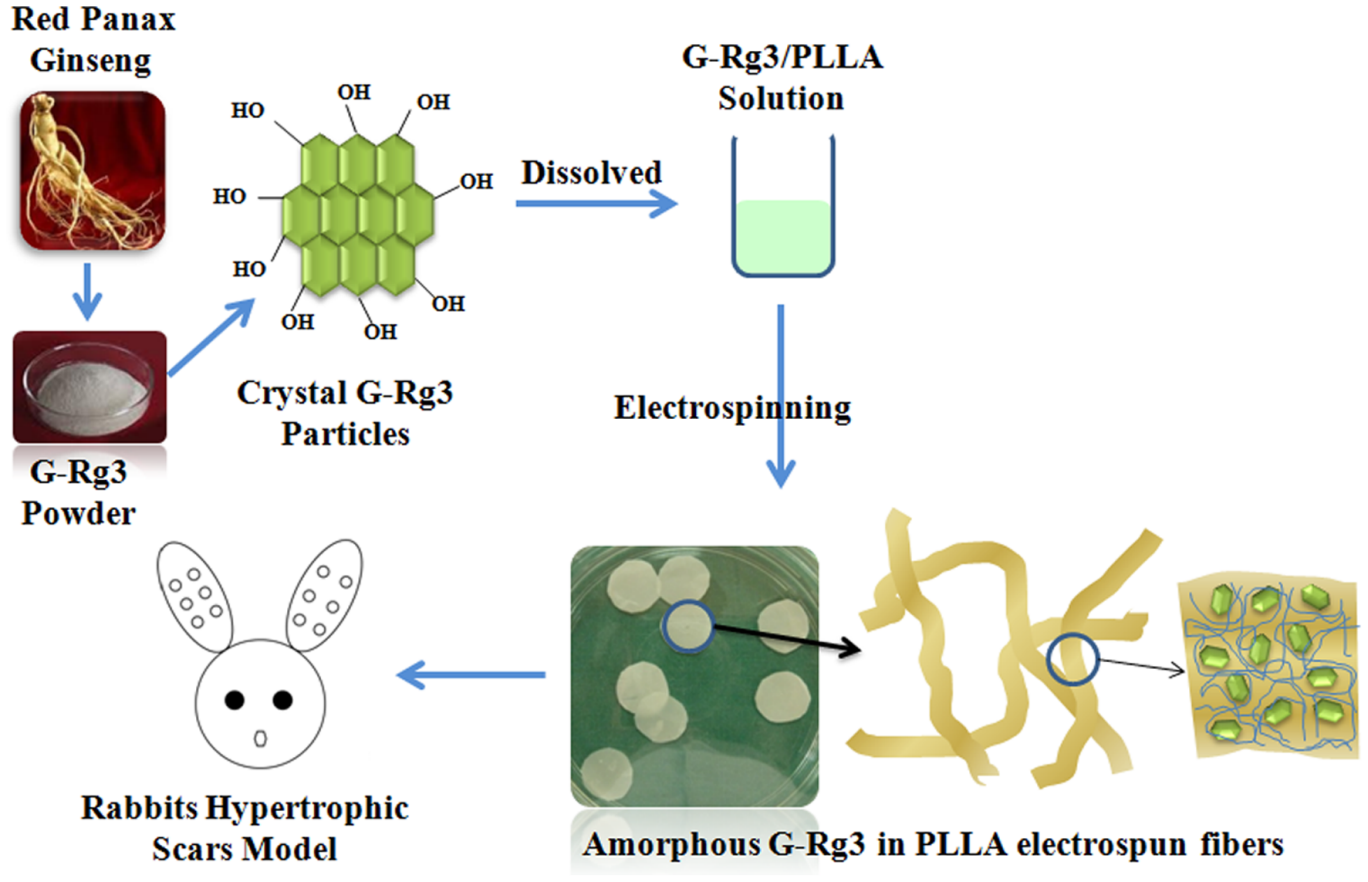
Amorphous state G-Rg3 in PLLA electrospun fibrous scaffolds for inhibiting hypertrophic scar formation.

Therefore, in this study, G-Rg3 and Poly(l-lactide) (PLLA) were selected for preparation of G-Rg3/PLLA electrospun fibrous scaffolds for inhibiting hypertrophic scar formation (Figure 1). The crystallization of G-Rg3 encapsulated in the electrospun PLLA matrix was characterized. The release behavior of G-Rg3 from PLLA matrix was evaluated and the effects of different amounts of G-Rg3 on the release pattern were studied. In addition, the matrix degradation behaviors of electrospun G-Rg3/PLLA fibers were investigated. Cell attachment and proliferation of fibroblasts seeded on the G-Rg3 loaded PLLA fibrous matrices were also examined, and *in vivo* studies were carried out to evaluate the ability of G-Rg3 loaded PLLA mats to reduce hypertrophic scar formation.

## Materials and Methods

### Materials

Poly(l-lactide) (PLLA, Mw = 50 kDa, Mw/Mn = 1.61) was purchased from Jinan Daigang Co. (Jinan, China). Ginsenoside Rg3 (G-Rg3) was obtained from Fusheng Pharmaceuticals Inc. (Dalian,China). 1,1,1,2,2,2-hexafluoro-2-propanol (HFIP) was purchased from Sigma-Aldrich (Saint Louis, MO). All chemicals and solvents were of reagent grade and purchased from Guoyao Regents Company (Shanghai, China).

### Electrospinning

20 mg, 60 mg and 100 mg G-Rg3 was dissolved in 2 g HFIP, respectively, and 1 g PLLA was dissolved in 2.5 g dichloromethane. The electrospinning solutions were prepared by mixing G-Rg3/HFIP solution and PLLA/dichloromethane solution. PLLA fibrous mats with different amounts of G-Rg3 (20 mg G-Rg3 in PLLA-2%, 60 mg G-Rg3 in PLLA-6% and 100 mg G-Rg3 in PLLA-10%) were obtained by electrospinning. The electrospinning processes were performed as described previously [28]. Briefly, the electrospinning apparatus was equipped with a high-voltage statitron (Tianjing High Voltage Power Supply Co., Tianjing, China) with a maximal voltage of 50 kV. Flow rate of the polymer solution was controlled at 0.6 ml/h by a precision pump to maintain a steady flow from the capillary outlet. A grounded aluminum foil was used as a collector. The G-Rg3/PLLA solution was loaded in a 2 ml syringe attached to a circular-shaped metal syringe needle as the nozzle. The voltage for electrospinning was set as 15 kV and the tip-to-collector distance was fixed at 12 cm. The electrospun scaffolds were collected on the surface of the aluminum foil and vacuum dried at room temperature for 24 h.

### Characterization of Electrospfun PLLA Fibrous Scaffolds

The thickness and size of the fibrous scaffolds were measured with a micrometer, and their apparent density and porosity were calculated according to previous methods [23].

The morphology of fibrous scaffolds was observed by scanning electron microscopy (SEM, FEI Quanta 200, Netherlands). At least five images were taken for each scaffold sample and fiber diameter of scaffolds was measured from SEM images with 5,000× magnification. From each image, at least 20 different fibers and 200 different segments were randomly selected to generate an average fiber diameter using Photoshop.

To investigate the crystalline phase of electrospun fibrous scaffolds, samples (20×20 mm^2^) were analyzed with X-ray diffraction (XRD, Philips X’Pert PRO, The Netherlands) over the 2 theta range from 5° to 70° with a scanning speed of 0.35°/min (λ = 1.54060 Å). Differential scanning colorimetry (DSC) measurements were performed using a DSC equipment (Netzsch STA 449C, Bavaria, Germany). The samples were analyzed in perforated and covered aluminum pans under a nitrogen purge. Approximately 1 mg of samples was heated from 25 to 150°C with a heating rate of 10°C/min.

For mechanical property tests, electrospun fibrous scaffolds were punched into small strips (70.0×7.0×0.6 mm^3^) and soaked in DMEM cell culture medium for 24 h before being tested. Uniaxial tensile tests were performed using an all purpose mechanical testing machine (Instron 5567, Norwood, MA) and the stress-strain curves of fibrous scaffolds were constructed from the load deformation curves recorded at a stretching speed of 0.5 mm/s (n = 5). From the stress-strain curves, Young’s modulus, tensile strength, and elongation at break of the scaffolds were obtained.

Surface wettability of the modified and unmodified electrospun PLLA scaffolds was evaluated by measuring water contact angles (WCA), which were measured at room temperature with a Kruss GmbH DSA 100 Mk 2 goniometer (Hamburg, Germany) followed by image processing of sessile drop with DSA 1.8 software.

### In vitro Loading and Release of G-Rg3

Loading efficiency of G-Rg3 in the PLLA fibrous scaffolds was determined by extracting the drug from fiber samples. In brief, a known amount of fibers (ca. 50 mg) were completely dissolved in 500 µl of acetonitrile, which was then added into 1.5 ml methanol to obtain a turbid liquid. The liquid was then centrifuged, and the supernatant was collected for detecting the G-Rg3 amount by high-performance liquid chromatography (HPLC). The extraction efficiency was calibrated by adding a certain amount of G-Rg3 into a polymer solution along with the same concentration as above and extracted by the same process.

For evaluating the *in vitro* release behavior of G-Rg3, the G-Rg3/PLLA fibrous scaffolds were first punched into small squares (2×2 cm^2^) with a total mass of ca. 50 mg, which were immersed in 20 ml of 154 mM phosphate buffered saline (PBS, pH 7.4), containing 0.02% sodium azide as a bacteriostatic agent. The suspension was kept in a thermostated shaking water bath (Taichang Medical Apparatus Co., Jiangsu, China) with a shaking speed of 100 cycles/min at 37°C, and the shaking speed was determined according to the references^24^. At predetermined time intervals, 5.0 ml of the release buffer was removed for analysis and 5.0 ml of fresh PBS was added back for continuing incubation.

The structure integrity and amount of released G-Rg3 were determined by HPLC with a UV detector set at 203 nm (Waters 2695 and 2487, Milford, MA) using fresh G-Rg3 as a standard. C18 stationary phase with a 5µm particle size and column dimensions of 4.6 × 150 mm was used at 30°C, and the mobile phase consisted of acetonitrile and distilled water (60/40 vol %) at a flow rate of 1.0 ml/min. For standard samples with a concentration of 0, 5, 10, 20, 30 and 40 µg/ml, a linear correlation (*γ* ^2^ = 0.9989) was determined between the absorption strength and G-Rg3 concentration. The percentage of the released drug in triplicate samples was then calculated based on the initial weight of the drug incorporated in the electrospun scaffold.

### *In vitro* Degradation of Fibrous Scaffolds

The degradation of G-Rg3/PLLA fibrous scaffolds was evaluated by measuring the molecular weight change and the mass loss. G-Rg3/PLLA fibrous scaffolds were accurately preweighted (ca. 100 mg each) and added to 50 ml of PBS (pH 7.4), containing 0.02% sodium azide as a bacteriostatic agent. At predetermined time intervals, samples were rinsed with distilled water to remove residual buffer salts, and dried to constant weight in a vacuum desiccator. The mass loss was determined gravimetrically by comparing the dry weight remaining at a specific time with the initial weight. The molecular weight of scaffolds was determined by gel permeation chromatography (GPC, waters 2695 and 2414, Milford, MA) using polystyrene as a standard. The column used was a Styragel HT 4 (7.8×300 mm). The mobile phase was consisted of tetrahydrofuran (THF) using a regularity elution at a flow rate of 1.0 ml/min.

### Cell Culture on Scaffolds

Human hypertrophic scar fibroblasts (HSFs), kindly provided by National Tissue Engineering Center of China (Shanghai, China) as a gift, were isolated from human hypertrophic scars obtained during the hypertrophic scar excision operation by sequential trypsin and collagenase digestion according to the method established by Kim et al [29]. HSFs were cultured in DMEM containing 10% FBS, penicillin (100 units/ml) and streptomycin (100 ug/ml) (Sigma) in a humidified incubator at 37°C with 5% CO_2_. Culture media were replaced every three days. The second to fourth passages of HSFs were used in this study.

Electrospun fibrous scaffolds of around 800 µm thickness were cut into small disks with a 5 mm diameter. Both sides of the disks were sterilized by ultraviolet irradiation in a laminar flow hood for 2 h and pre-wetted with cell culture medium. 50 µl of HSFs suspension (3×10^4^ cells/ml) were seeded on the surfaces of disks and placed in 96-well culture plates (Costar, Corning, NY, USA). Then, cell-seeded scaffolds were incubated at 37°C with 5% CO_2_ for 4 h to allow cells diffuse into and adhere to the scaffolds before the addition of 100 µl of culture medium into each well. The cell-seeded scaffolds were replenished with fresh medium every 3 d.

### Cell Morphologies and Proliferation

Samples were harvested on day 1, 3, 6 and 9 after seeding, washed with PBS twice, and fixed with 4% glutaraldehyde for 2 h at 4°C. After being rinsed with distilled water for 3 times, the samples were dehydrated through a series of graded ethanol solutions. The dry specimens were sputter coated with gold and the cell morphologies on the fibrous scaffolds were observed by SEM. Cell proliferation was determined by CCK-8 assay (Cell counting kit-8, Dojindo, Kumamoto, Japan). The CCK-8 reagent is a faint red substance that produces a saffron yellow formazan product when incubated with viable cells. Therefore, the level of CCK-8 into formazan can reflect the level of cell metabolism. On day 1, 3, 6 and 9 after incubation, 15 µl of CCK-8 was added to each well, and incubated at 37°C for 2.5 h according to the reagent manufacture’s instruction. 100 µL aliquot of incubated medium was pipetted into a 96-well plate and a microplate reader (Thermo labsystems, Helsinki, Finland) was used to detect the absorbance of each well at 450 nm. At each time point, three samples were analyzed for each group.

### *In vivo* Animal Test in a Rabbit Model

The hypertrophic scar model was established using New Zealand white rabbits. The anti-scarring properties of the G-Rg3/PLLA fibrous scaffolds on full-thickness wounds were studied in a New Zealand white rabbit hypertrophic scar model. The New Zealand white rabbits were subject to round full-thickness skin excisions by a method described previously [30]–[32]. 15 New Zealand White rabbits were purchased from Animal Center of Shanghai (Chinese Academy of Science, China). An Institutional Review Committee of Shanghai Jiao Tong University School of Medicine approved all animal study protocols. The New Zealand white rabbits were weighing 4.5–5 kg and were kept under standard conditions. Animals were anesthetized with an intramuscular injection of ketamine (22.5 mg/kg) and xylazine (2.5 mg/kg) followed by isoflurane gas through tracheal intubation. Six full-thickness wounds were created down to the cartilage on the ventral side of each ear using a 10 mm punch biopsy, carefully avoiding the central ear artery and marginal ear veins. The cartilages were meticulously nicked without full dissection, as the latter would cause undirected granulation tissue and epithelial ingrowth resulting in noninterpretable histology. Epidermis, dermis, and perichondrium were thoroughly removed using a dissecting microscope. Complete removal of the perichondrial layer is mandatory as it delays epithelialization for 8–14 days. Occasional bleeding is treated by manual compression and electro-cauterization should only be considered in unappeasable bleedings as it increases the risk of wound necrosis with subsequent alteration of the wound-healing. Each two wounds were separated by at least 1.3 cm apart. A 3 mm-round-skin undermining was then made on each wound to enable the embedding of the electrospun membranes. Totally 180 wounds were created and analyzed. Wounds were divided into five groups, namely group A, group B, group C, group D, and group E, each containing 36 wounds. Each group consisted of the wounds on both lateral and medial sides of the ear.

The electrospun fibrous scaffolds of PLLA, PLLA-2%,/PLLA-6% and PLLA-10% were cut into round pieces (with a diameter of 1.2 cm), which fitted the wounds. They were then sterilized by electron-beam irradiation using linear accelerator with a total dose of 80 cGy before treatment. In group A, B, C and D, each wound was covered with PLLA-2%, PLLA-6%, PLLA-10% and PLLA electrospun fibrous scaffolds, respectively. While in group E, the wounds were not covered and healed naturally as the control. Dry gauze was covered on the wound area, and the gauze edge was sutured to the skin around the wound area.

Wounds were observed daily and photographs were taken on day 30 and 90 postoperatively. Animals were sacrificed on day 30 and 90, and scars were harvested with a 0.5 cm margin of surrounding unwounded tissue. Totally 180 excision wounds were studied in this step.

### Histology

All the tissue samples were cut in a full-thickness manner from the wound sites, and bisected through the maximum point of scar hypertrophy on visualization and palpation. For histological analysis, the harvested samples were fixed in 4% formaldehyde/PBS solution at 4°C, dehydrated with a graded series of ethanol and embedded into paraffin. The sectioned samples with a thickness of 5µm were then stained by hematoxylineeosin (H&E), and visualized by an optical microscope.

### Masson’s Trichrome Staining for Collagen Fibers and Blood Vessels

Paraffin-embedded sections (4 mm) were mounted on glass slides and stained for collagen fibers and blood vessels using Masson’s Trichrome (collagen stains green). For each wound, 5 randomly chosen fields of dermis were photographed at 400× magnification.

### Statistical Analysis

Data used in the experimental methodology were obtained at least in triplicates. These data are also expressed as mean values with standard deviations (SD). One way ANOVA with the Tukey’s post-hoc test was used to discern the statistical difference between groups. A probability value (p) of less than 0.05 is considered as statistically significant.

## Results

### Characterization of Electrospun PLLA Scaffolds

**Figure 2:**
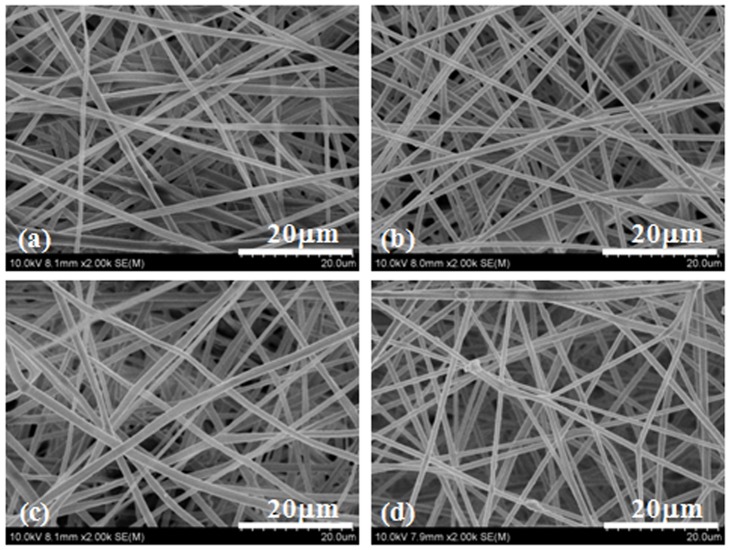
SEM images of electrospun fibers. PLLA fibers (a), PLLA-2% (b), PLLA-6% (c) and PLLA-10% (d) with 0%, 2.0%, 6.0% and 10.0% Rg3, respectively.

Figure 2 shows the fibrous morphology of the electrospun PLLA scaffolds. It can be seen that there are no beads in the fibrous structure and the fibers are uniform in size, randomly interconnected. The diameters of fibers were 1.08±0.46, 1.34±0.62, 1.21±0.37 and 1.41±0.42 µm for PLLA electrospun fibrous scaffolds with drug entrapment of 0% (PLLA), 2% (PLLA-2%), 6% (PLLA-6%) and 10% (PLLA-10%), respectively (Table 1).

**Table 1:** Characteristics of electrospun PLLA fibrous scaffolds with G-Rg3 (n = 3).

Sample	Average diameters (µm)	Mass per unit area (mg/cm^2^)	Porosity (%)	Shrunken (%)
PLLA	1.08±0.46	1.35±0.18	64.5±5.7	5.03±0.41
PLLA-2%	1.34±0.62	1.28±0.16	68.4±4.4	3.25±0.29
PLLA-6%	1.21±0.37	1.25±0.14	64.1±6.4	2.86±0.44
PLLA-10%	1.41±0.42	1.22±0.16	70.1±4.9	2.17±0.35

Porosity and shrinkage of electrospun PLLA fibrous scaffolds were characterized and the results were summarized in Table 1. The drug entrapment did not significantly affect the porosity of electrospun PLLA fibrous scaffolds, since there is no significant difference in the porosity among PLLA fibrous scaffolds (64.5%) and G-Rg3/PLLA fibrous scaffolds (64.1%–70.1%). Furthermore, the drug entrapment amount had no significant effect on the shrinkage of the fibrous scaffolds. After being incubated in PBS at 37°C for 1 week, electrospun PLLA fibrous scaffolds showed a dimensional shrinkage of 5.03%, and G-Rg3/PLLA fibrous scaffolds showed a dimensional shrinkage of 2–3%.

**Figure 3:**
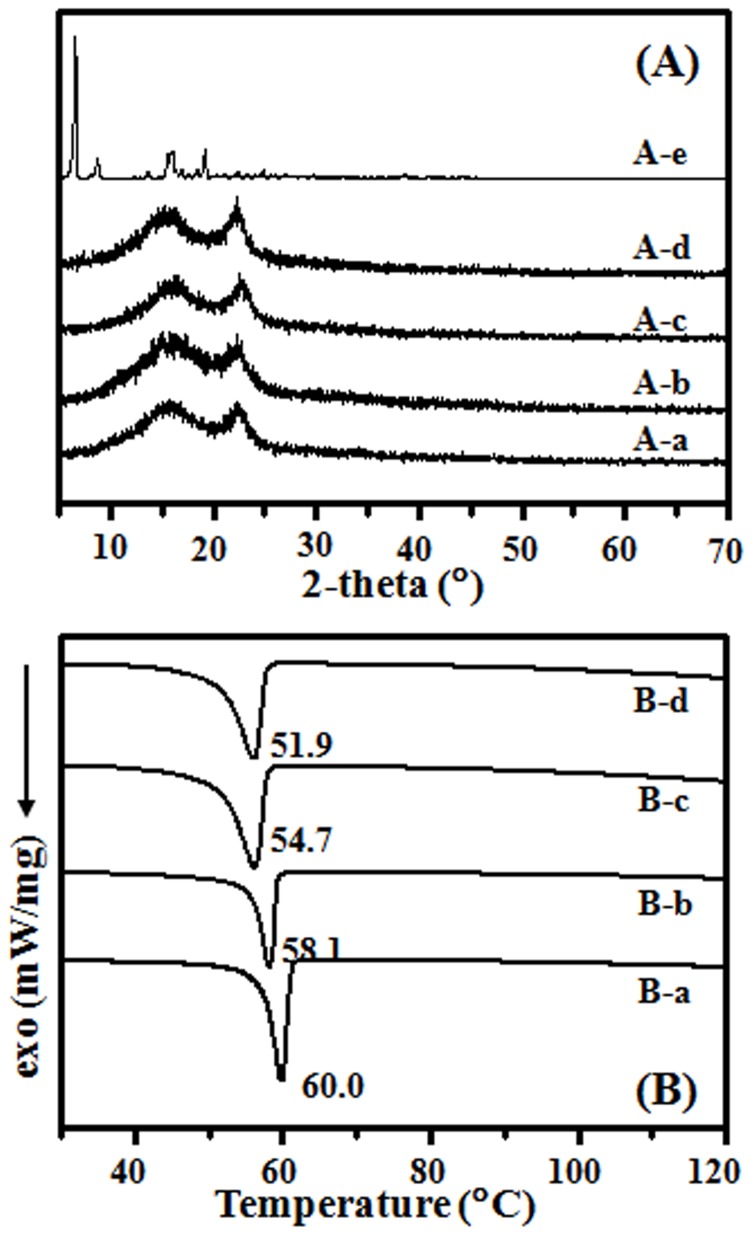
X-ray diffraction profiles (A) and DSC analysis (B) of electrospun fibers. PLLA fibers (A-a and B-a), PLLA-2% (A-b and B-b), PLLA-6% (A-c and B-c), PLLA-10% (A-d and B-d) and Ginsenoside Rg3 (A-e).

The crystalline state of incorporated drug in the electrospun fibers were examined by XRD, and Figure 3A summarizes the results. Characteristic crystalline peaks of electrospun PLLA fibrous scaffolds were observed at (110) and (131) in Figure 3A–a, b, c and d samples. Pure G-Rg3 was crystalline with major peaks at 2θ = 6.3, 8.4, 15.6 and 18.9° (Figure 3A–e), respectively, while no G-Rg3 peak was found in drug loaded PLLA scaffolds even in the sample of PLLA-10% (Figure 3Ab, c and d). The crystalline peak of G-Rg3 was not detected in all PLLA electrospun scaffolds containing G-Rg3, suggesting that G-Rg3 existed in amorphous form in PLLA- G-Rg3 scaffolds.

To determine the effect of drug inoculation on the thermodynamic behavior, DSC traces of electrospun scaffolds with and without G-Rg3 were recorded, which are shown in Figure 3B. The glass transition temperatures (*Tg*) of electrospun PLLA, PLLA-2%, PLLA-6% and PLLA-10% fibers (Figure 3B–a, b, and c) were 60.0, 58.1, 54.7 and 51.9°C, respectively. By incubating drug into electrospun polymeric fibers, the small molecular drug acted among the molecular chains and made molecular chains moving easily, which led to a lower Tg. The glass transition enthalpy was 6.89 J/g and 12.48 J/g for electrospun scaffolds of PLLA-10% and PLLA, respectively. The drug inoculation led to irregular alignment of polymer chain, which led to a slight decrease in the transition enthalpy.

To clarify the effects of the drug on the surface properties of electrospun fibers, water contact angles (WCAs) of electrospun fibrous scaffolds were measured, and the WCAs of electrospun fibrous scaffolds were 128.6±4.7, 132.9±5.8, 137.4±3.4 and 139.1±4.6 for electrospun PLLA fibers, PLLA-2%, PLLA-6% and PLLA-10%, respectively. The possible reason for this phenomenon is that the G-Rg3 is hydrophobic which contribute to the hydrophobic characteristic of the scaffolds incorporated with higher amount of drugs. However, statisticallythere was no significant difference between these samples.

To clarify the effects of G-Rg3 on the mechanical properties of electrospun PLLA scaffolds, the strain-stress measurements on wet scaffolds were conducted and the stress/strain results are shown in Table 2. G-Rg3/PLLA fibrous scaffolds showed higher modulus but lower tensile strength and elongation at break than PLLA scaffolds. These data also showed a decrease of the maximum mechanical tensile strength and maximum elongation with the increase of the G-Rg3 content in G-Rg3/PLLA fibrous scaffolds and an increase of the maximum modulus of elasticity with the increase of the G-Rg3. However, the statistical analysis indicates no significant difference of the mechanical properties between the G-Rg3/PLLA and PLLA fibrous scaffolds (P>0.05) (Table 2).

**Table 2:** Stress/strain data for the electrospun PLLA fibers, PLLA-2%, PLLA-6% and PLLA-10% (n = 3).

Sample	Maximum tensile strength (Mpa)	Maximum elongation (%)	Modulus of elasticity (MPa)
PLLA	3.95±0.42	53.45±4.28	30.25±3.26
PLLA-2%	3.84±0.37	50.18±4.64	31.18±3.75
PLLA-6%	3.56±0.34	44.35±3.94	34.95±3.56
PLLA-10%	3.38±0.31	39.42±4.35	37.95±4.11

### *In Vitro* Drug Release Behavior of PLLA Fibrous Scaffolds

**Figure 4:**
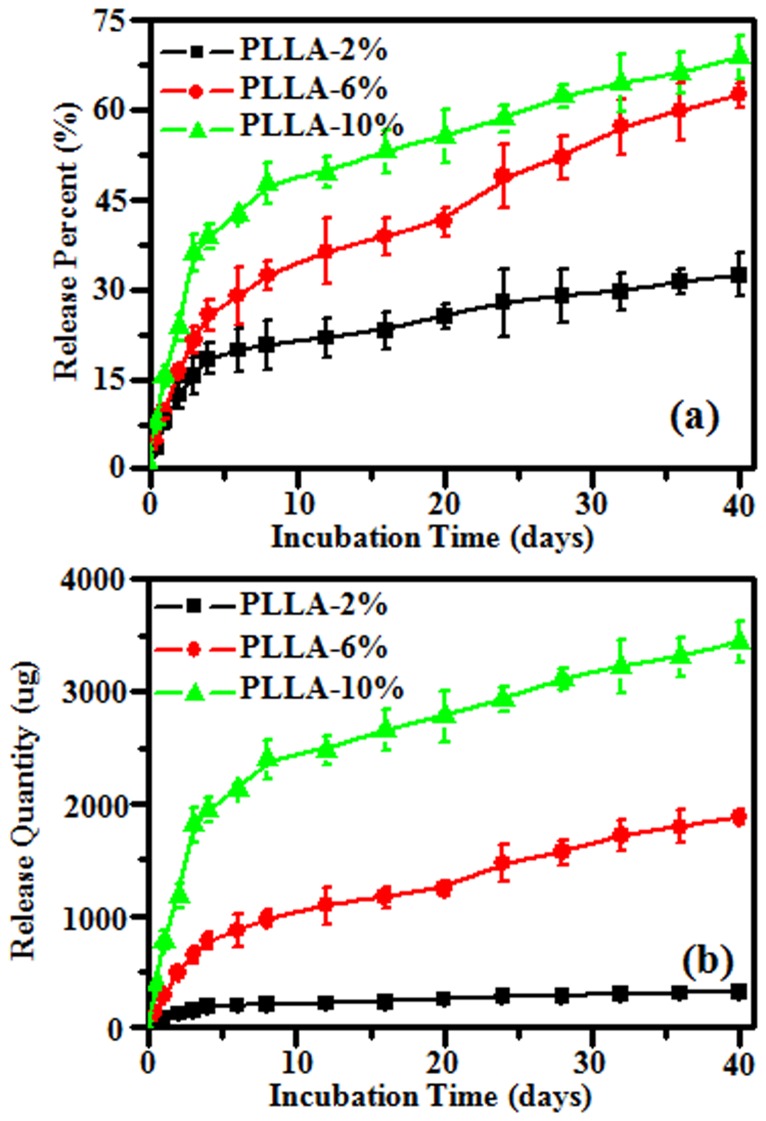
In vitro Rg3 release cumulative percentage (A) and cumulative quantity (B) from electrospun fibers after incubating in PBS at 37°C.

The drug release profiles of PLLA electrospun scaffolds with G-Rg3 loadings of 2.0, 6.0 and 10.0% were evaluated in buffer solutions and the results are summarized in Figure 4A and B. The release kinetics of G-Rg3 can be illustrated in two stages in Figure 4A: an initial burst release followed by a constant slow release. All electrospun scaffolds exhibited a linear release behavior during the first stage. Nearly zero-order release profiles were observed during the first 48 h, and the total amount of release was 18, 26, and 42% during this stage for scaffolds with drug entrapment of 2.0, 6.0 and 10.0%, respectively. After the initial burst release, a sustained release profile was observed for PLLA electrospun scaffolds with 6% and 10% of the G-Rg3 loading, and over 50% of drug released during 30 days incubation. However, a less significant release from PLLA electrospun fibers with 2% G-Rg3 loading was detected with the initial burst release of 18% followed by sustained release of additional 15% during 30 days incubation.

*In vitro* Rg3 release cumulative quantity from 50 mg G-Rg3/PLLA electrospun fibrous scaffold was summarized in Figure 4B. The release profiles of all the fibers were same to the cumulative percentage, and including an initial burst release and a constant slow release. There was about 324, 1878 and 3445 µg G-Rg3 release in a 40-day release period as seen in the fibrous scaffold for 2.0, 6.0 and 10.0% of drug entrapment, respectively. The cumulative release quantity of G-Rg3 could be calculated from different amount of electrospun fibrous scaffolds, and these data could be used as reference data for animal tests.

The structural integrity of G-Rg3 in the electrospun fibers after electrospinning and incubation in the release medium was determined by HPLC in the current study. The results show same maximum absorbance peak at 203 nm, and same elution time of 4.78 min for pure G-Rg3, G-Rg3 in electrospun fibers, and G-Rg3 released into the release medium. Thus, it can be concluded that the chemical structure of the released G-Rg3 appears to be intact after electrospinning processing and releasing. The G-Rg3 was found to have poor aqueous solubility, and the solubility of the pure G-Rg3 powder was only 3.2 µg/ml in PBS. In contrast, the G-Rg3 solubility from PLLA-2%, PLLLA-6% and PLLA-10% scaffolds were 10.2, 14.7 and 19.4µg/ml respectively, which was improved for near 6 times in PLLA-10% sample as compared with the pure G-Rg3 powder (Table 3), suggesting that electrospun fibers could effectively improve dissolubility of G-Rg3.

**Table 3:** G-Rg3 concentration after soaking pure G-Rg3 and drug loaded fibrous scaffolds in PBS (n = 3).

	G-Rg3	PLLA-2%	PLLA-6%	PLLA-10%
Highest content(µg/ml)	3.24±0.43	10.22±1.78	14.71±1.91	19.43±1.84

### *In vitro* Degradation Behavior of PLLA Fibrous Scaffolds

**Figure 5:**
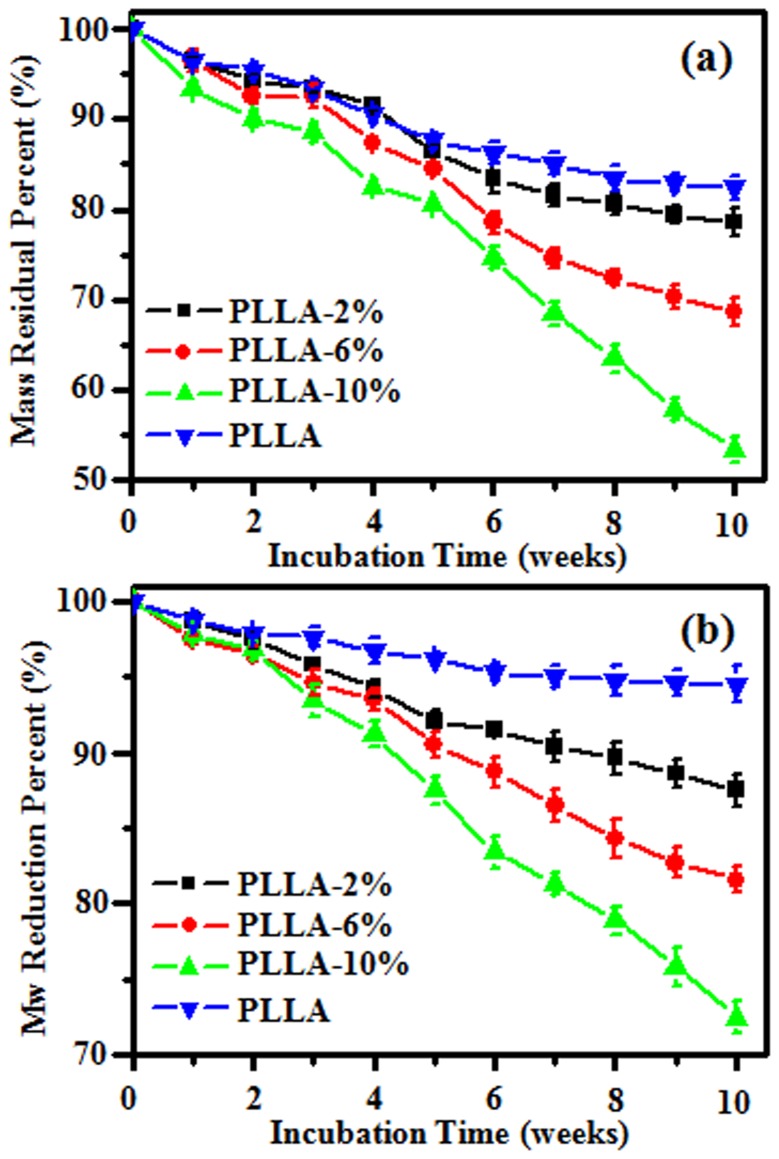
Residual mass percent (a) and molecular weight reduction (b) of electrospun fiber scaffolds after incubating in PBS at 37^o^C.

The degradation behaviors of PLLA, PLLA-2%, PLLLA-6% and PLLA-10% electrospun fibrous scaffolds were determined in buffer solutions with regard to the mass loss of the fiber matrix, and the molecular weight reduction of the matrix polymer. The mass loss and molecular weight change of electrospun fibrous scaffolds during incubation are summarized in Figure 5. There were 17.1% and 6.5% of mass loss and molecular weight reduction during 10 week incubation of electrospun PLLA fibers, respectively. But a faster degradation rate was detected for electrospun G-Rg3/PLLA fibers, and there were 21.4, 31.3 and 46.6% of mass loss and 12.5, 18.4 and 27.5% of molecular weight reduction for PLLA-2%, PLLLA-6% and PLLA-10%, respectively. As shown in Figure 5, the entrapment of G-Rg3 into the fibers matrix speeded up the degradation of the matrix polymer comparing to pure PLLA fibers.

### Human Hypertrophic Scar Fibroblasts Culture on PLLA Fibrous Scaffold

**Figure 6:**
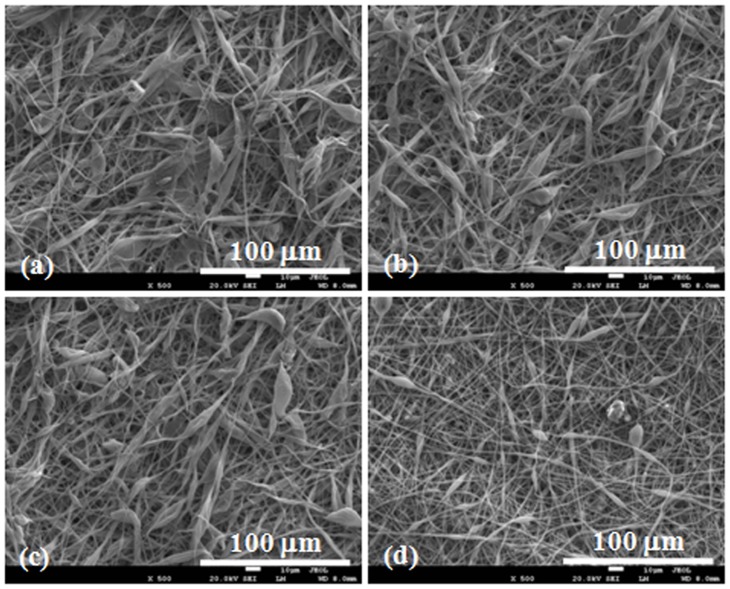
SEM images of HSFs growth on the electrospun fibrous scaffolds after 9 d culture. (a) PLLA fibers, (b) PLLA-2%, (c) PLLA-6% and (d) PLLA-10%.

HSFs cell morphology after being cultured on the fibrous scaffolds for 9 days was observed by SEM, and the images are shown in Figure 6. Cells bridged the electrospun fiber filaments and were thoroughly hybridized with the fibrous network on the PLLA fibers and PLLA-2%. By day 7, few cells merely covered the surface of the PLLA and PLLA-2% fibrous scaffolds (Figure 6a and b). It was very hard to find HSFs cells on PLLA-6% and PLLA-10% fibrous scaffolds after 9 days of culture (Figure 6c and d).

**Figure 7:**
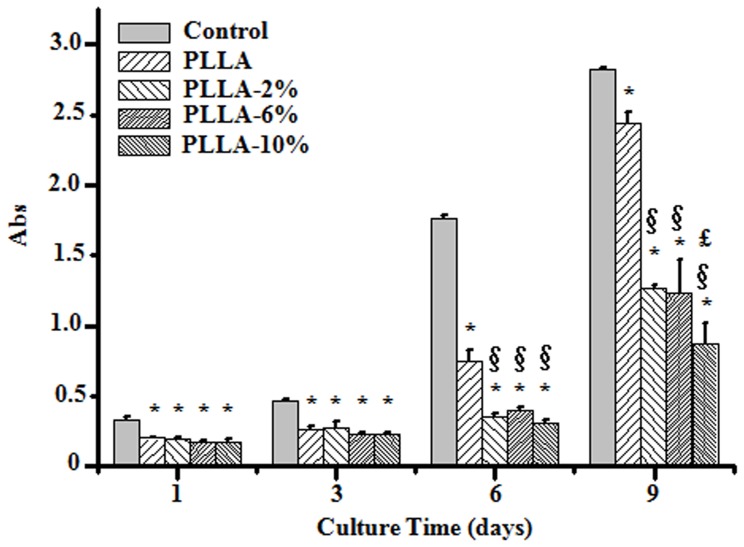
CCK-8 assay of HSFs growth after 1, 3, 6 and 9 d culture. TCP was set as control. *P<0.05 compared with control. ^§^P<0.05 compared with PLLA. ^£^P<0.05 compared with PLLA-2% and PLLA-6%.

The HSFs cell proliferation was measured using a colorimetric CCK-8 assay. The cell culture plate (TCP) was used as control. All results are summarized in Figure 7. Higher HSFs cell proliferation rates were observed on control and electrospun PLLA scaffolds as compared to those on G-Rg3/PLLA scaffolds, and this phenomenon became obvious after the cells were cultured for 6 and 9 days (P<0.05). After 9 days culture, the cell proliferation rate on PLLA-10% scaffolds was significantly lower than those on PLLA-2% and PLLA-6% scaffolds (P<0.05), which indicated that increase of G-Rg3 amount in PLLA fibers resulted in further inhibition of HSFs cell proliferation.

### Gross Observation and Histology of Scar Healing Process

**Figure 8:**
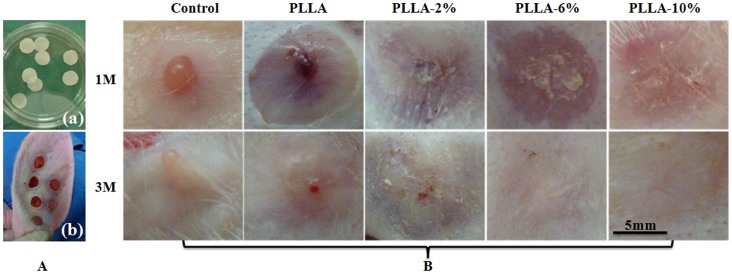
Gross observation of scar hyperplasia harvested on 1 and 3 months of each group. (A) Implanted fibrous scaffolds (a) and rabbits hypertrophic scars model (b); (B) Gross observation of scar hyperplasia treated with blank, PLLA fibers, PLLA-2%, PLLA-6% and PLLA-10% for 1 and 3 months after operation.

As shown in Figure 8, at one month, the control group showed a visibly raised, contracted, reddish tissue resembling early scar formation (Figure 8A). The wounds treated with G-Rg3/PLLA fibrous scaffold (PLLA-2%, PLLA-6% and PLLA-10%) also showed some evidence of HS, but the scars were less raised and less contracted as compared to those on the PLLA scaffolds. The scar turned flatter as the portion of G-Rg3 in PLLA became higher, and it is flattest in PLLA-6% and PLLA-10% group. However, the wounds in the PLLA fibrous scaffold group and control group showed obvious evidence of HS with raised and reddish tissues in the wound area.

At three months, the hypertrophic scars in control group and PLLA group turned relative white, but were still raised and mild contracted. The scars in group PLLA-2%, PLLA-6% and PLLA-10% also became much whiter at three months than at one month. The scar was mild raised in PLLA-2% group; while in PLLA-6% and PLLA-10% group, the scars were difficult to discern, as they were almost as flat as the skin, and their color were close to the skin nearby.

**Figure 9:**
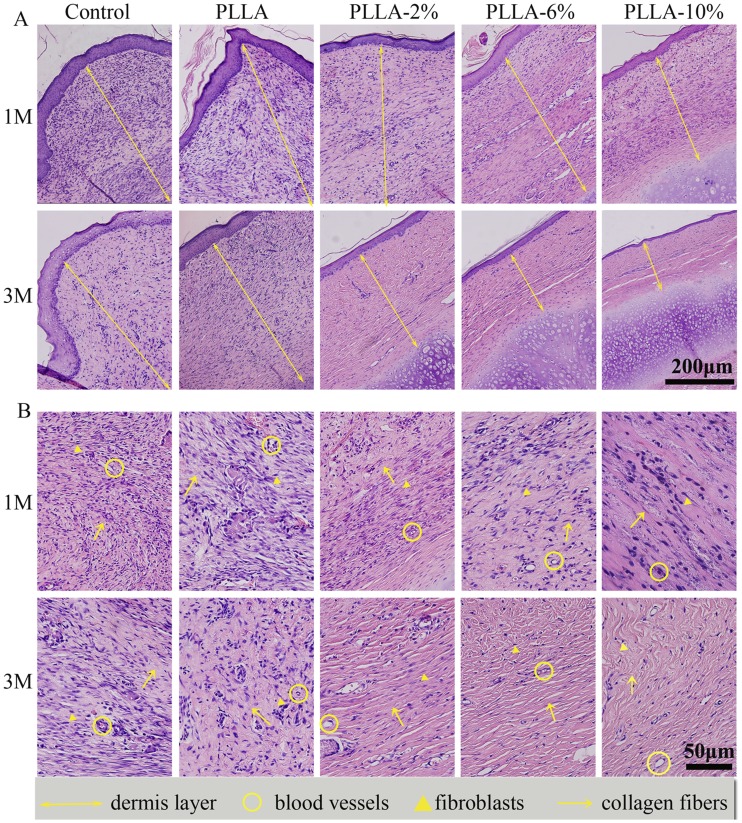
H&E staining of sections of scar hyperplasia at 1, and 3 months after wounding. The upper two rows show H&E staining at 100×. The lower two rows show H&E staining at 400×. The dermal layer of the control scars and the PLLA-treated scars were much thicker than PLLA-2%, PLLA-6% and PLLA-10% electrospun fiber-treated groups at 1 M and 3 M. The dermal layer was thinnest in the PLLA-10%- treated group at 1 M and 3 M. The yellow arrow indicates collagen, the yellow triangle indicates fibroblasts and the yellow circle indicates blood vessels.

**Figure 10:**
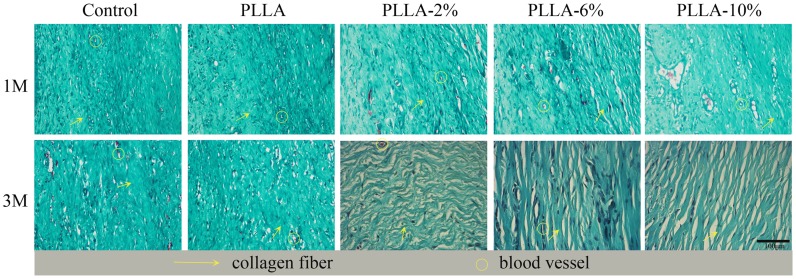
Masson’s staining of sections of scar hyperplasia at 1, and 3 months after wounding at 400 magnifications. The arrows represent the collagen fibers, and the circles represent the vessels. The bar represents for 100 µm.

Histological analyses were performed to assess the anti-scarring properties of the different G-Rg3/PLLA fibrous scaffolds on full-thickness wounds, and the results are present in Figure 9 and Figure 10. At day 30, there were typical infiltrations of fibroblasts in all groups, with different distributions and density. In the group PLLA and control, the dermis layer was significantly thickened. Abundant fibroblasts and thick, tight collagen fibers were seen in the whole layer, with a badly organized arrangement. Abundant microvessels were also observed in this layer. In comparison, the fibroblasts were mostly distributed in the upper dermis layer in group PLLA-2%, PLLA-6%, and PLLA-10%, and the collagen fibers were assembled in parallel. The density of fibroblasts, collagen fibers, and microvessels were all reduced in group PLLA-2%, PLLA-6%, and PLLA-10%.

At day 90, the mature phase of hypertrophic scar, the number of fibroblasts were greatly decreased, but still infiltrated in the whole dermis layer in group PLLA and control. The collagen fibers were still in a badly organized arrangement in group control and in group PLLA. The dermis in group PLLA-2%, PLLA-6%, and PLLA-10% were thinner than that in group PLLA and control, and the density of fibroblasts and collagen fibers were greatly decreased. The collagen fibers all assembled in a parallel way. The density of fibroblasts and collagen fibers were smallest in group PLLA-10%. In general, all the G-Rg3/PLLA electrospun fibers showed positive effect on HS, and PLLA-6% and PLLA-10% showed better effect than PLLA-2%. However, no significant difference was observed between PLLA-6% and PLLA-10% groups.

## Discussion

### Co-dissolved PLLA and G-Rg3 Solution for Electrospinning

G-Rg3 has methyl groups and hydroxyl groups on its backbone, which on one hand makes G-Rg3 itself hydrophobic and on the other hand brings G-Rg3 a polyanion property [33]. The poor solubility of G-Rg3 under organic solvent makes it difficult to design its pharmaceutical dosage form [16], [34], and the extremely poor solubility of G-Rg3 under physiological conditions makes it be challenging to regulate G-Rg3’s release rate from polymer matrix with the aim to maintain suitable drug concentration within a therapeutic window for enough exposure time [35]. Therefore, it is important to find a G-Rg3 drug delivery system, which can be effectively carry and release G-Rg3. PLLA polymers have been extensively investigated for drug delivery applications due to their biocompatibility, biodegradability, and absorbability [36]. In addition, PLLA electrospun fibrous scaffolds have also been widely investigated as drug delivery system, due to the large surface area, high porosity and flexility. It has been reported that PLLA electrospun scaffolds could control drug release and effectively improve the therapeutic window for enough exposure time [37]. Therefore, we proposed that PLLA electrospun scaffolds might also be a good delivery system for G-Rg3. To obtain PLLA exlectrospun scaffolds, there are two technologies: melting electrospinning and solution electrospinning. However, it is found that blending G-Rg3 with PLLA at a highly miscible level is very difficult to achieve for two main reasons: (1) G-Rg3 has a high melting point of 315°C, and PLLA will decompose before G-Rg3 melting. Therefore, it is impossible to blend G-Rg3 with PLLA by the melting electrospinning processing technique though PLLA is thermoplastic. (2) So far, to our knowledge, there are no shared common solvents in which both G-Rg3 and PLLA can be well dissolved for solution electrospinning. G-Rg3 can only be dissolved in very low amounts in methanol and dimethyl sulphoxide solutions because of its specific structure [38], while PLLA can normally be dissolved in some organic solvents such as chloroform, dichloromethane, and tetrahydrofuran. Some studies have reported that HFIP, a volatile solvent (bp 59°C) with very potent solvency, can be used as a solvent for PLLA [39]. In this study, we found that HFIP was also a good solvent for G-Rg3. When the G-Rg3/HFIP solution was added into PLLA/dichloromethane solution, a transparent homogeneous solution could be obtained, which was suitable for fabrication of fibrous scaffolds by electrospining. Therefore, HFIP and dichloromethane were an ideal mixing organic solvent system for both G-Rg3 and PLLA. This mixing solvent allowed full extension of the polymer chains and complete evaporation of solvent during the electrospinning process for the formation of electrospun fibers.

### Improving Solubility of G-Rg3 in Electrospun Fibers

Some literatures have reported that G-Rg3’s crystallinity affects its dissolvability in physiological medium since the crystalline phase is less accessible to the water molecules [11]. Therefore, changing its crystalline state into amorphous form might be an effective way to improve the solubility of G-Rg3. In our study, SEM and XRD results indicated that the G-Rg3 carried in PLLA electrospun fibers was in amorphous state. As it is well known that G-Rg3 is a drug in crystalline form, the electrospinning process of PLLA-G-Rg3 fibrous scaffolds obviously changed G-Rg3’s crystal state. During the electrospinning process, the large surface area associated with ultrafine fibers allowed fast and efficient solvent evaporation, which gave dissolved drugs limited time to recrystallize and amorphous state was maintained. Our results indicate that electrospinning of PLLA–G-Rg3 mixtures is not only an effective method for one-step incorporation of G-Rg3 in fibrous scaffolds but also allows the successful change of the G-Rg3 from a crystallized state into an amorphous state. The obtained results are in accordance with the data for other electrospun drug/polymer systems [40]–[42]. Since there is no crystal lattice energy to be overcome for dissolution, the amorphous state facilitates the dissolution of G-Rg3 from the PLLA elextrospun fibrous scaffolds, which demonstrated by 6-fold higher concentration of G-Rg3 released from the PLLA electrospun fibrous scaffolds as compared to that from the pure G-Rg3 particles. Moreover, the amount of the released G-Rg3 in the physiological medium could be further altered by adjusting the G-Rg3 incorporation amount in PLLA electrospun fibers (Figure 4 A and B).

### The Effect of G-Rg3 on Preventing the Hypertrophic Scar Development

HS is a dermal fibroproliferative disorder that often occurs following deep trauma, severe burn injury, and surgical incision. Normally, the characteristics of HS are cell over-proliferation and collagen over-deposition. The HSFs cell is the main cell for producing collagen matrix [4], so the HSFs cell proliferation is correlated with HS formation [5]. Our *in vitro* results have demonstrated that electrospun G-Rg3/PLLA fibrous scaffolds significantly inhibited HSFs cell proliferation, and this inhibitory effect is dependent on the incorporated G-Rg3 amount at low concentration. However, when the G-Rg3 amount in the electrospun scaffolds is higher than 6%, no further improvement on the inhibitory effect was observed suggesting that 6% might be the optimal dose for inhibiting cell proliferation. Our *in vivo* results also confirm the advantages of the G-Rg3 on prohibiting the scar development. Compared with the blank and PLLA electrospun fibrous scaffolds, the G-Rg3/PLLA electrospun fibrous scaffold showed significant improvements in terms of dermis layer thickness, HSFs proliferation, collagen fiber density and microvessels, revealing that the incorporation of the G-Rg3 in the electrospun fibers prevented the HS formation. It is noticed that no scaffolds were observed in the 1 M and 3 M histology sections. Some previous studies have shown that the polyester biomaterials degraded faster *in vivo* than *in vitro* [43], [44]. Therefore, it is possible that the partially degraded scaffolds and polymer fibers with a diameter less than 1–2 µm might be washed out during the staining procedure. Furthermore, the *in vivo* results also confirmed the observation of *in vitro* study, which showed that PLLA-6% and PLLA-10% had better inhibitory effect on scar formation than PLLA-2%, while no significant difference between PLLA-6% and PLLA-10% groups. Therefore, the 6% G-Rg3 in PLLA electrospun fibers might be the optimal composition of the electrospun scaffolds for inhibiting HS.

### Conclusions

Herein the G-Rg3/PLLA electrospun fibrous scaffold was prepared and applied to reduce hypertrophic scar formation. The G-Rg3/PLLA electrospun fibrous scaffold was successfully fabricated using HFIP/dichloromethane as co-solvent in a one-step process, which allows the successful incorporation of the G-Rg3 in the fibers in an amorphous state and easy dissolution from the fibrous scaffolds. The amount of G-Rg3 released from the PLLA electrospun fibrous scaffolds was near 6-fold higher than that from the pure G-Rg3 particles. The G-Rg3/PLLA electrospun fibrous scaffold showed the initial burst release in first 2 days and then exhibited a gradual release for more than 30 days. The *in vitro* culture of HSFs on the PLLA-G-Rg3 scaffolds revealed that the G-Rg3 encapsulated fibrous scaffolds may have the potential ability to inhibit HSFs cell proliferation. In the *in vivo* experiments, the G-Rg3/PLLA electrospun fibrous scaffolds of PLLA-6% and PLLA-10% were found to be able to inhibit HS formation in the HS model of rabbit by decreasing thickness of formed dermis and numbers of proliferative cells and collagen fibers. These findings suggest that the G-Rg3/PLLA electrospun fibrous scaffolds may be used for inhibiting HS and restoring the structural and functional properties of wounded skin.
